# Developing a training programme for midwives and maternity support workers facilitating a novel intervention to support women with anxiety in pregnancy

**DOI:** 10.1186/s12884-022-04996-2

**Published:** 2022-08-25

**Authors:** Kerry Evans, Helen Moya, Marissa Lambert, Helen Spiby

**Affiliations:** 1 University of Nottingham, School of Health Sciences, Queen’s Medical Centre, Nottingham, NG7 2HA UK; 2 Moya CBT, Loughborough, Leicestershire UK; 3 With-you Consultancy Ltd, St. Helens, Merseyside, UK

**Keywords:** Anxiety, Perinatal, Training, Midwives, Mental health

## Abstract

**Background:**

The RAPID-2 intervention has been developed to support women with symptoms of mild-to-moderate anxiety in pregnancy. The intervention consists of supportive discussions with midwives, facilitated discussion groups and access to self-management materials. This paper reports the development of a training programme to prepare midwives and maternity support workers to facilitate the intervention.

**Methods:**

Kern’s six-step approach for curriculum development was used to identify midwives and maternity support workers training needs to help support pregnant women with anxiety and facilitate a supportive intervention. The stages of development included feedback from a preliminary study, stakeholder engagement, a review of the literature surrounding midwives’ learning and support needs and identifying and supporting the essential process and functions of the RAPID intervention.

**Results:**

Midwives’ reported training needs were mapped against perinatal mental health competency frameworks to identify areas of skills and training needed to facilitate specific intervention mechanisms and components. A training plan was developed which considered the need to provide training with minimal additional resources and within midwives’ scope of practice. The training plan consists of two workshop teaching sessions and a training manual.

**Conclusion:**

Future implementation is planned to include a post-training evaluation of the skills and competencies required to fully evaluate the comprehensive programme and deliver the RAPID-2 intervention as planned. In addition, the RAPID-2 study protocol includes a qualitative evaluation of facilitators’ views of the usefulness of the training programme.

## Background

Each year in the UK approximately 750,000 women use midwifery services, and 14% will experience symptoms of anxiety [1]. Anxiety disorders are associated with postnatal depression, low birthweight, premature birth and developmental and behavioural problems in children [2–6]. For women with mild-moderate anxiety, psychological support may help reduce anxiety and prevent an escalation of symptoms [1]. However, services have not been developed or rigorously tested in pregnancy. Midwives are ideally positioned to support women’s mental health during pregnancy and the postnatal period [7]. It is recognised that maternity care has focused on physical wellbeing and greater attention to support emotional health is required [8]. Perinatal mental health is a priority area identified in the National Health Service long term plan [9] which aims to provide an additional 24,000 women each year with access to specialist perinatal mental healthcare. However, studies have highlighted that midwives lack confidence to support women with mental health experiences and report barriers in screening and supporting perinatal mental health needs.

The RAPID intervention is the first midwife-led intervention to be evaluated for pregnant women with symptoms of mild to moderate anxiety and the first to include midwifery support workers (MSWs) as co-facilitators. Preliminary work was conducted to design and develop the intervention and a small scale study was completed 2016 in an NHS Trust in England (RAPID-1). The design and development work, including stakeholder and service user engagement, conceptual and theoretical frameworks and outcome measures has been reported in a previous paper [10].

**Fig. 1:**
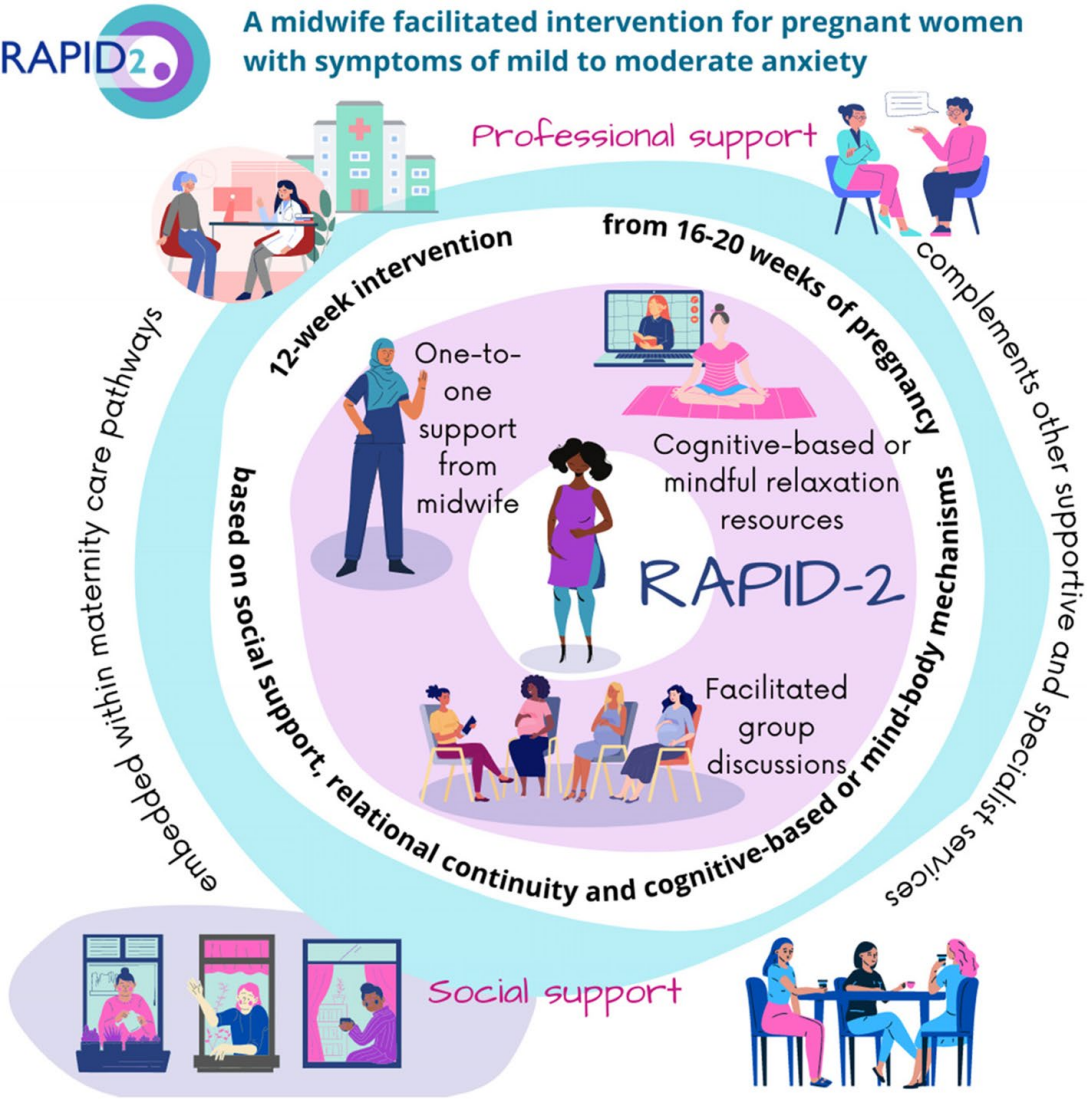
Components of the RAPID-2 Intervention

The RAPID intervention comprises three components: 1) One-to-one pre-group introductory meeting with the midwife facilitator; 2) Facilitated group discussion sessions. Midwife and maternity support workers (MSWs) help to initiate discussions by asking women about their feelings and wellbeing during the week. The role of facilitator is to cultivate and model the opportunities for peer support through shared learning and experiences rather than leading discussions. Facilitators also help women introduce discussion topics they may find difficult to introduce themselves. Facilitators will also support women’s wellbeing and signpost and support women to access specialist or supportive services where appropriate; 3) A choice of self-help materials for women to access between groups. The choice of materials is based on service user preferences and relevance in a UK healthcare context (Fig. 1).

Preliminary work demonstrated the intervention could be integrated within routine maternity care and women and midwife and MSW facilitators felt they had benefitted from participating. It was important for the proposed intervention that, in addition to the local maternity care structures, wider maternity care policy and strategy were considered. This was particularly relevant as the intervention development was being conducted during the publication of new national maternity care policy and would need to be operational in both existing and future maternity care contexts [11]. In addition, the involvement of midwives and MSWs to facilitate the intervention was motivated from the wider midwifery care literature which stressed the need to strengthen the role of the midwife in promoting women’s mental and emotional wellbeing [12]. Thus, developing an intervention which could be delivered within midwives’ scope of practice [13], with minimal additional resources and which could be integrated into midwifery services was of particular importance.

The RAPID-2 study is planned to be conducted in 2022 and aims to test the feasibility of a midwife facilitated intervention for pregnant women with symptoms of mild to moderate anxiety (https://fundingawards.nihr.ac.uk/award/NIHR300376). To enhance the fidelity of an intervention, it is essential facilitators receive appropriate training to prepare them to deliver the intervention as planned [14].

This paper reports the stages of development of a bespoke training package to prepare midwives and MSWs to facilitate the RAPID-2 intervention.

## Methods

The training programme was developed following Kern’s six-step approach for curriculum development [15]. Kern’s six-step approach was selected as it is used extensively in healthcare education and has been reported beneficial to assist, organise and summarise key issues specific to the development of clinical and professional skills curricula [16–18]. A team consisting of the lead midwife researcher (KE) and two mental healthcare professionals and educators (HM, ML) developed the training programme with feedback and advice from a study advisory group (comprising maternity care researchers, midwifery managers, a consultant clinical psychologist) and service users from a maternity research public involvement group.

### Problem identification and general needs assessment

The study advisory group raised concerns that training midwives to deliver CBT and mindfulness-based interventions would be intensive, with training usually taking 1 year or more to complete. It was suggested that mind body and cognitive behavioural approaches could be delivered through supported use of self-help resources. The advisory group suggested ways in which women could be supported by midwives within midwives’ current scope of practice. A brief training programme was suggested to prepare midwives to facilitate peer groups and act as a resource to the women. It was agreed that a midwifery support worker could be trained to co-facilitate the intervention. There were no readily accessible training courses focused on facilitating interventions for pregnant women or supporting women with mild-to-moderate anxiety in pregnancy.

A brief training intervention for groups of midwives which focused on advanced communication skills education (providing woman-centred care, active listening, discussing sensitive issues, communication strategies) was reported to increase midwives’ comfort and competence to identify and care for women with psychosocial issues during the postnatal period [19]. A very brief training intervention (1/2 day) for health visitors, using a combination of case studies and vignettes was found to empower health visitors to identify perinatal mental health concerns and promote confidence [20].

#### Training to recognise, address and support women’s mental health in pregnancy

A literature review was conducted to identify midwives training and support needs to support women’s mental health and wellbeing.

Midwives can feel uncomfortable as they feel they lack expertise to respond appropriately to women’s perinatal mental health (PMH) concerns and can be unsure of the actions they should take [21]. Although midwives have reported good knowledge of the prevalence, consequences and causes of PMH problems, they felt ill-equipped to support women and many did not feel prepared to respond to the PMH needs of women [21, 22]. Midwives have reported a lack of training to prepare them for a role in perinatal mental healthcare, in particular asking questions about mental health, supporting women from different cultures, and responding appropriately to women’s disclosures of mental health issues [21–23]. Fear of causing women further distress or offence and midwives’ personal discomfort in discussing mental health have all been reported as barriers to midwifery PMH care provision [23]. Midwives’ self-identified training needs include information about the types of mental health concerns, non-pharmacological management options and skills to help women recognise and cope with stress [12, 24]. Clear pathways of care, local guidelines, access to specialist advice and educational resources have also been identified as areas which needed to be strengthened in order to improve the role of the midwife in mental health care provision [12, 23–26]. Midwives have identified a need for effective and on-going educational interventions to be introduced along with clinical supervision, mentoring and opportunities for debrief [26].

There is little published literature on the role of the MSW in supporting women’s mental and emotional health. The Maternity Support Worker Competency, Education and Career Development Framework [27] propose four domains of practice including providing supportive care for women and families. This highlights the MSWs role in developing supportive relationships with women, having knowledge and understanding of mental health concerns and referring or escalating concerns to an appropriately registered practitioner.

### Targeted needs assessment

The targeted training needs assessment focused on midwives’ reported needs to support women’s wellbeing (as reported in stage 1) and facilitators’ training needs to support the mechanisms of change underpinning the RAPID intervention.

#### Theoretical approach to the RAPID intervention

The theory underpinning RAPID includes: 1) Social support theory; 2) Therapeutic relation theory; 3) Mind-body approaches; 4) Cognitive-behavioural mechanisms [10]. It is considered that the intervention will promote positive change in women’s anxiety symptoms through 1. developing collaborative relationships which aim to promote women’s choice and control over their care. 2. receiving support from healthcare professionals (HCPs) who both understand women’s individual needs and can also help them access services; 3. accessing support and learning from other women who have experienced / are experiencing similar feelings or situations; 4. developing strategies to help women develop an awareness of their thought processes and learn techniques to improve the way they cope with anxiety.

#### Supportive role

The RAPID intervention will require midwives and MSWs to provide care, support and guidance within their current scope of practice. Midwives and MSW facilitators will not be expected to deliver therapeutic content, but to provide care to support women’s wellbeing and signpost and support women accessing specialist services where appropriate.

#### Facilitative role

Midwives require additional training and support as they transition to group facilitators [28, 29]. In maternity care, the role of the HCP in breastfeeding support groups has been reported to “normalise or counteract extreme views and help women to distinguish between fact, anecdote and myth” [30] (page 143). In a group based antenatal care study, women appreciated midwives contributing their expertise in antenatal care and helping to address topics women found difficult to introduce [31].

#### Perinatal mental health competency frameworks

The following established competency frameworks were accessed to: 1) consider the scope of midwifery practice [13], and; 2) highlight aspects of perinatal mental healthcare which would be useful to prepare the midwife and MSWs in facilitating RAPID-2:

- The Perinatal Mental Health Curricular Framework [32]
- The Competency Framework for Professionals Working with Women who have Mental Health Problems in the Perinatal Period [33]
- Caring for Women with Mental Health Problems: Standards and Competency Framework for Specialist Maternal Mental Health Midwives [34]

Individual competencies within these frameworks, which were considered useful and relevant for facilitating the intervention were mapped into domains, considering the methods of training and highlighting potential useful resources (Table 1).

**Table 1:** Training domains, resources and method of training for intervention facilitators and co-facilitators

Training Domains	Training resources and supporting documents Reading lists, on-line resources and summaries of key points for inclusion in the training manual / workshop
Overview of perinatal mental health: prevalence; symptoms for common PMH concerns; clinical guidelines	Reference documents and resources • Clinical guidelines: mental and perinatal mental health CG192 [35] • Health Education England: e-learning package: Introduction to perinatal mental health [36]
Factors which impact on mental wellbeing in pregnancy and associated outcomes	• NHS Education for Scotland. E-learning module: Understanding maternal mental health [37] • Risk factors for PMH [38] • Perinatal outcomes associated with PMH concerns [39]
Identification of the symptoms and risk factors for anxiety and other mental health disorders	• Clinical guidelines: mental and perinatal mental health CG192 [35] • Identifying anxiety in pregnancy [40] • Women’s views on PMH screening [41, 42] • Midwives’ views on PMH screening [24, 26, 43]
Supporting women’s mental health in pregnancy	• Good practice guides [34, 44] • Clinical guidelines: mental and perinatal mental health CG192 [35] • Professional standards [13]
Signposting and referring to other supportive services	• Local NHS Trust maternity mental health procedures and guidelines • Local perinatal mental health supportive services and referral pathways: PMH teams, charities, IAPT services (referral pathways) • NHS Education for Scotland. E-learning module: Maternal mental health, the woman’s journey [37]
Overview of therapeutic and mind-body approaches	• Cognitive behavioural approaches for worry, anxiety and coping • Mindfulness and relaxation techniques • Clinical guidelines: mental and perinatal mental health CG192 [35] • Active listening skills [45]
Peer support mechanisms and peer groups	• Good practice guides [46, 47] • Perinatal mental health peer support [48–51]
Self-help help resources	• Supporting women to complete self-management tools based on cognitive behavioural and /or mind-body approaches [52]
Practice within legal and professional policy frameworks	• Professional standards [13] • Antenatal and postnatal mental health: clinical management and service guidance [35]

### Goals and objectives

Specific objectives for the training were developed by identifying the essential process and functions of components of RAPID to maintain the overall study objectives while enabling facilitator flexibility within different contexts. The RAPID-2 study is considered a complex intervention as it: 1) includes several interacting components; 2) is sensitive to the context in which it is delivered; 3) has a causal chain linking the intervention to outcomes; 4) has a range of possible outcomes [53]. Due to the complexity of the intervention, it was important to develop a training programme which provides facilitators with the knowledge and skills to support the theory underpinning intervention components and enable facilitators to have a degree of flexibility of the intervention delivery and techniques [54]. Incorporating flexibility can: 1. enable facilitators to use their creativity and meet the individual needs of the women; 2. provide opportunities early in the training to experiment while receiving supervision and feedback from trainers; 3. allow early opportunities for learning the importance of tailoring the intervention to meet the needs of the woman in different settings [28, 54]. Table 2 outlines the components, functions and training requirements of each component of the intervention.

**Table 2:** Intervention components and training objectives

Principle of the intervention	Types of standardisation		Training objectives
	By component	By function	Skills and awareness
To provide access to individual support for pregnant women with symptoms of mild to moderate anxiety	Facilitate individual time for women to speak with the facilitator. Access to a private room away from the groups. Provide information about further supportive services which women can access.	Promote a safe supportive environment where women feel confident to discuss their feelings and concerns.	Develop skills in fostering therapeutic and collaborative relationships. Acknowledge that women may have concerns about disclosing their symptoms. Identify ways to support women to disclose their symptoms and seek support. Discussing the importance of working within professional guidelines and identify other supporting services and referral pathways.
To enable pregnant women to provide and receive peer support and build social support networks	Hold four group meetings, one every 2-weeks. Midwife and MSW facilitators work in collaboration with women to develop group agendas and ground rules.	Promote a safe supportive environment where women can talk about topics important to them. Encourage women to share experiences and offer and receive support from other group members.	Develop an understanding of the beneficial mechanisms which underpin peer support: experiential knowledge, social learning and comparison, help-seeker / help-provider. Explore the role of the facilitator in peer groups through active learning approaches. Work within professional guidelines. Develop strategies for formally ending groups.
To guide women in accessing and completing self-help resources to improve their symptoms of anxiety	Facilitators to provide an overview of the self-help resources and encourage women to feedback about their progress with the resources.	Self-help resources can help develop coping mechanisms for symptoms of anxiety. Women should be offered a choice of approaches and formats.	During the training, facilitators will familiarise themselves with the self-help material and acknowledge the mechanisms which underpin each resource (relaxation, mind-body and cognitive behavioural approaches)

### Educational strategies

An integrative review by Brunero et al. [55] reported pedagogical styles for mental health education programmes included a mixture of didactical and experiential learning. Experiential mechanisms ask HCPs to reflect on their experiences from practice. Scenario-based learning, role modelling, rehearsal, reflection and feedback are commonly used experiential approaches used in mental health education [56]. Trainees are more committed to training that they perceive will enhance their current practice, using real examples promotes self-regulated learning and consideration of the context of practice. Mental health training for healthcare professionals should not be too far removed from the trainees’ knowledge base yet different enough to enthuse, engage and facilitate commitment to the programme [56].

While most training programmes are delivered face-to face there is a growing need to deliver far reaching resources using on-line technology, particularity in the current COVID-19 pandemic. Midwives have reported to prefer attending study days to receive PMH education as opposed to online delivery which may be reflective of interactive approaches to skills development [26]. Other studies have highlighted that midwives welcome flexible training options including online packages, seminars and workshops [22].

#### Pilot work: feedback from the preliminary study

Before training, midwife and MSW facilitators initially felt uncomfortable when women disclose mental health concerns. Their concerns were addressed through practical skills and guided role-play activities, learning techniques to manage potentially challenging situations and signposting women to further supporting services. All facilitators felt they had developed an understanding that they were not required to provide all of the answers to address women’s concerns [57]. The training for the preliminary study was delivered by two different training providers, each with different areas of expertise. This caused some initial confusion for the midwives and MSWs regarding the different options to support women with anxiety: a cognitive based therapy approach and a peer-based approach. It was recommended that a more cohesive programme should be developed, with trainers identifying the inter-play between the intervention components and how they can provide increased choice, empowerment and options for women to improve their experiences of anxiety. Facilitator training workshops for the preliminary study was conducted over 3 days (over a two-week period). Feedback from all four facilitators suggested workshops could be reduced to 2 days and the training manual could be developed into an interactive workbook to encourage facilitators to reflect on how different supportive techniques could be used to respond to situations which may arise [54]. MSW co-facilitators felt they had benefitted from attending the same training workshops as the midwives. This helped to identify their role and acknowledge the role boundaries and responsibilities between the midwives and MSWs.

### Implementation

Following the evaluation of the current literature, educational approaches and feedback from stakeholder group, the facilitator training plan was designed and approved by the mental health professional trainers, midwifery professionals and researchers. The training plan consists of workshop teaching, discussion and exercises and a training manual with information and self-complete exercises (table 3). Two training workshops were planned with training-free days in between to focus on self-care and reflect on their learning. Recruitment of facilitators is planned for summer 2022. To support recruitment, discussions have taken place between the researcher and senior midwives and managers in the study sites with initial agreement to recruit two midwife and two MSWs per study sites to receive the training and facilitate the RAPID intervention.

**Table 3:** Training plan for rapid-2 facilitators

**Components**	**Delivery**	**Essential skills and knowledge**	**Useful knowledge**	**Pre-group exercises**
**Introducing RAPID-2**	**Training manual**	Intervention components		
1. Common anxiety concerns in the perinatal period	**Training manual** Additional e-learning resources: NHS elearning for healthcare: perinatal mental health [36] • Introduction to Perinatal Mental Health 1 • Introduction to Perinatal Mental Health 2 • Perinatal Mental Health in the Antenatal Period		• Incidence rates • Overview of the types of anxiety • Vulnerability to anxiety	Reflective exercise: Experiences of caring for women with anxiety in pregnancy. What did they share? How did they appear? What things made you concerned or reassured? How did they communicate their anxiety?
2. Midwives and maternity support workers role in supporting women’s mental health	**Training manual** Additional e-learning resources: NHS Education for Scotland: Maternal Mental Health [37] • Understanding maternal mental health • Maternal mental health, the woman’s journey	NICE recommendations: • GAD-2 screening • Support and treatment options for mild to moderate anxiety • Symptoms and risk factors for severe mental health concerns • Referral pathways [35]	• Barriers and facilitators to communicating anxiety concerns: • Self-awareness and avoidance • Stigma and fear of consequences • Self-comfort in discussing mental health	Reflective exercise: Experiences of caring for women with anxiety in pregnancy. How comfortable do you feel? What helps to start a conversation? What fears do women have talking about mental health?
3. Ways to support women with anxiety. An overview of different approaches	**Training manual** **Workshop day 1:** *Delivered by Mental Health Professional with specialist knowledge and experience of CBT and mindfulness approaches. Sessions contain formal teaching, group discussion and exercises.*	Overview of social support, therapeutic relation theory (collaborative therapeutic relationships) Overview of the principles of the Cognitive Behavioural Therapy model and Mindfulness, Problem Solving and Relaxation		
4. Supportive communication	**Training manual** **Workshop day 1 and 2:** *Delivered by Mental Health Professional with specialist knowledge and experience of CBT and mindfulness approaches. Sessions contain formal teaching, group discussion and exercises.*	Active listening skills Asking questions Body language		
5. Accessing other local supportive services				Reflective exercise: What services are available to support women’s mental health in the local area and how to access / referrer? • Specialist perinatal mental health teams • Psychological therapies • Charity and community and user-led organisations
6. Peer-based approaches for women with anxiety in pregnancy	**Training manual** **Workshop day 2:** *Delivered by Mental Health Professional with specialist knowledge and experience in peer-based approaches. Sessions contain formal teaching, group discussion, creative and listening exercises.*	• Fundamental principles of peer support • Safe environments • Building relationships • Sharing stories and emotions • Learning from each other	Evidence-base: peer support in pregnancy: reduce isolation; challenge idealised depictions; normalise concerns; challenge negative self-perception; feeling understood; sharing experiences and building self-confidence	Written exercise ‘Sharing stories’: explore facilitators personal boundaries for sharing their life stories in groups: what would / would not feel comfortable (own personal reflection) Exercise ‘Who am I’ poster: exploring strengths, skills and interest of facilitators – what facilitators contribute to groups
7. Working in groups and maintaining peer networks	**Training manual** **Workshop day 2:** *Delivered by Mental Health Professional with specialist knowledge and experience in peer-based approaches. Sessions contain formal teaching, group discussion, creative and listening exercises.*	• Role of the facilitator • Creating a safe space • Cultivating compassion • Supporting group dynamics • Challenging situations • Ending groups Proving one-to-one time: • Signposting and escalation	Roles and mechanisms within peer group approaches: • Social learning and social comparison • Help-seeker / help-provider	
8. Support for facilitators	**Training manual** **Workshop day 2:** *Delivered by Mental Health Professional with specialist knowledge and experience in peer-based approaches. Sessions contain formal teaching, group discussion, creative and listening exercises.*	• Facilitation within the scope of midwifery practice – accessing usual methods of support • Where to turn when facilitators have concerns (Research lead, PMA, managers and clinical leads)	Online resources: • Supporting others and looking after yourself [58, 59] • Better health ‘Mind Plan’ [60] • Your Personal Resilience Plan [61]	

### Evaluation and feedback

The effectiveness of mental health training programmes for healthcare professionals has been evaluated through pre-post training knowledge tests, attitudinal scales, clinical audit an self-report measures (perceived knowledge, confidence, efficacy and skills) [55].

Evaluation of the training programme plays an integral role in the RAPID-2 feasibility study, to assess the usefulness of the programme and preparing facilitators to deliver the intervention and identifying the optimal programme content and timing. Evaluation and analysis will be completed by the lead researcher (KE) and discussed with the study advisory group and engagement with service users. The plan is to evaluate the training at two time points: 1. A training evaluation questionnaire (delivered post-training); and 2: a qualitative evaluation of facilitators views of the usefulness of RAPID-2 training (delivered post-intervention). The Kirkpatrick model [62] of evaluation has been used to the identify the impact on the training programme across the four domains:

- Reaction (post-training questionnaire): the quality of the manual and workshops; participants’ overall satisfaction with the training.
- Learning (post-training questionnaire): awareness of the symptoms of anxiety in pregnancy; awareness of coping strategies for women with mild to moderate anxiety in pregnancy; awareness of other supportive services for women with anxiety in the local area; feeling prepared to deliver the intervention; feeling confident to talk to women about anxiety / support women with anxiety within current scope of practice / provide women with evidence-based information to support their wellbeing / recognise emergency situations and take appropriate actions
- Behaviour (post-intervention qualitative interviews with facilitators): ability to respond appropriately to women’s situations and concerns about anxiety; ability to support and guide the use of self-help resources; ability to facilitate and manage group discussions
- Results (post-intervention qualitative interviews with facilitators and participants): Women’s views on participating in the intervention; facilitators views on the wider benefits of the training on their practice

A process evaluation is planned which will follow the framework developed by Grant, et al. [63] to explore the delivery of the intervention, intervention fidelity, maintenance, context, unintended consequences and theory of change. Intervention fidelity will be established through a structured facilitators’ notes review including anonymised summaries of any individual discussions and the topics covered in the groups. Group sessions will be audio recorded (with consent) and anonymised data will be analysed to describe group content and assess fidelity between groups. Training and facilitator evaluation will inform further refinements required prior to conducting a definitive trial.

## Conclusions

The RAPID programme of research has developed an intervention manual, training materials, and workshops ready for testing in the RAPID-2 study. The RAPID-2 training intervention was developed through an iterative process, reflecting on facilitators’ feedback from the preliminary RAPID study. The use of the Kern’s six-step approach for curriculum development assisted the training programme development. The framework enabled established perinatal mental health competencies, evidence-based and theoretical approaches, educational strategies and midwives reported training needs to be synthesised to produce a comprehensive overarching training framework. The ultimate aim is to demonstrate the benefit of a midwife facilitated intervention which can be implemented with minimal resources into current models of maternity care and provide, timely support for women with mild to moderate anxiety symptoms, to prevent an escalation of symptoms and improve women’s ability to cope.

## Data Availability

All data generated or analysed during this study are included in this published article [and its supplementary information files].
